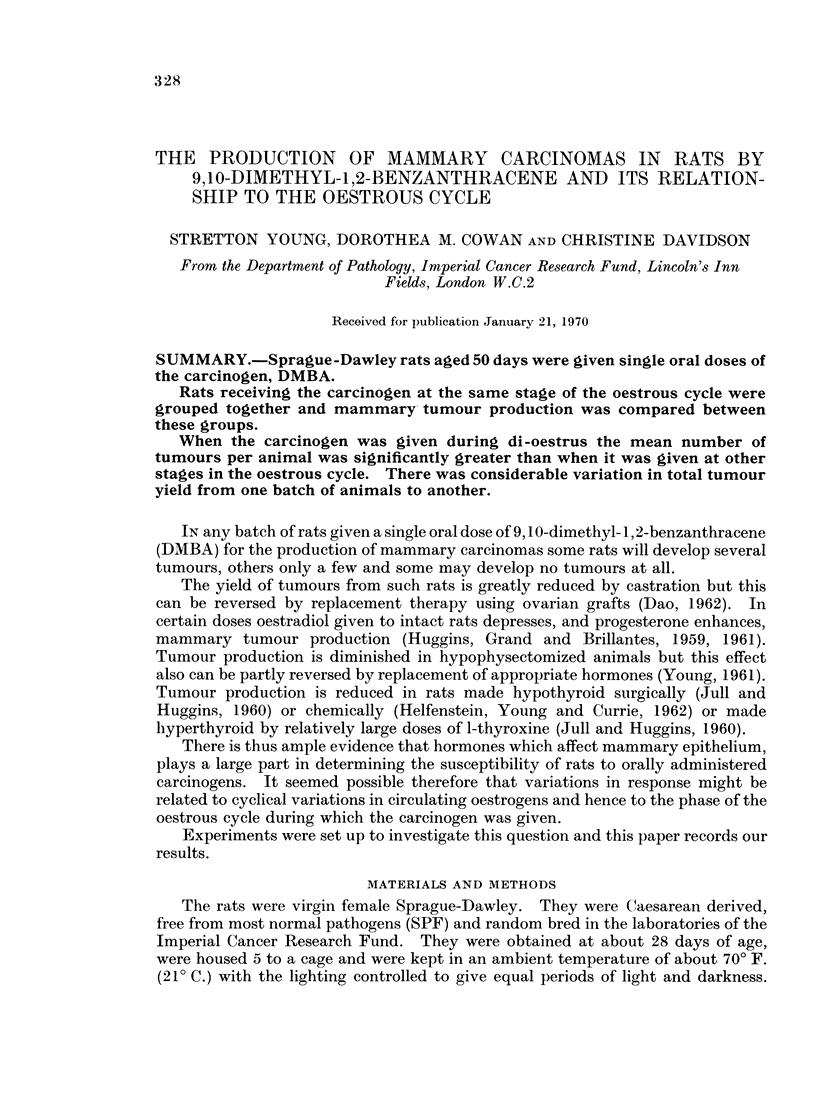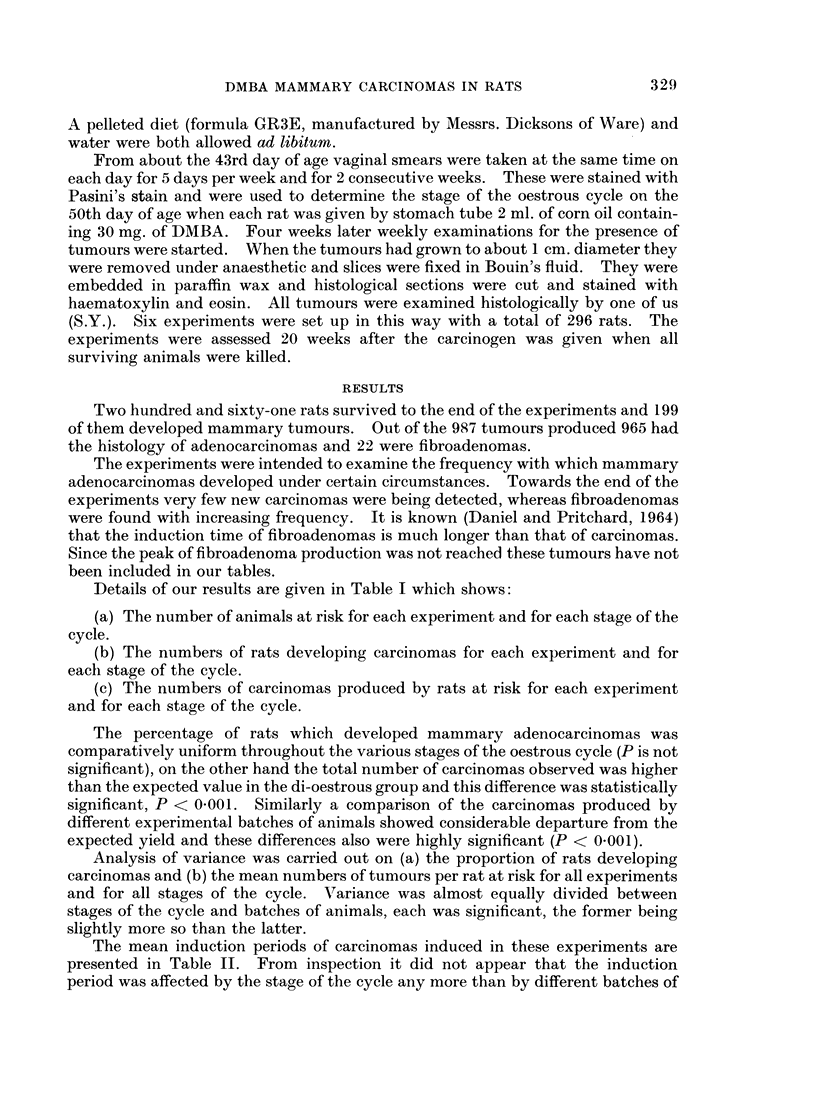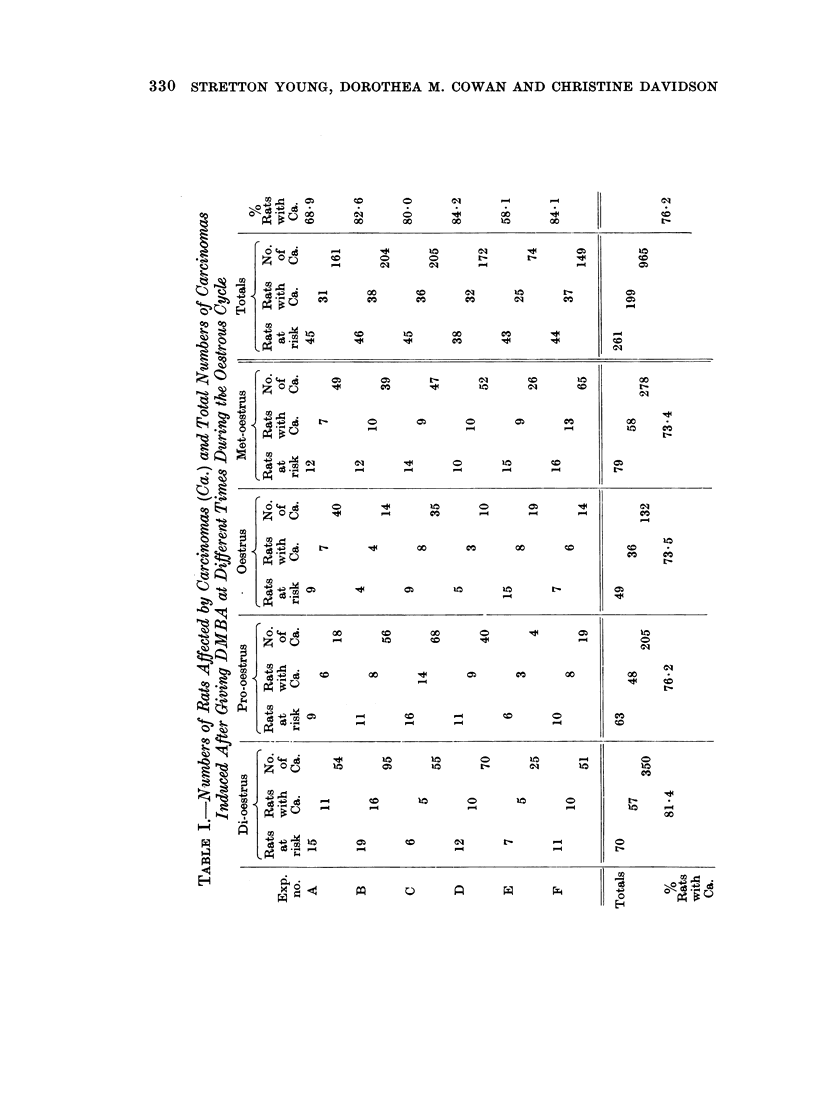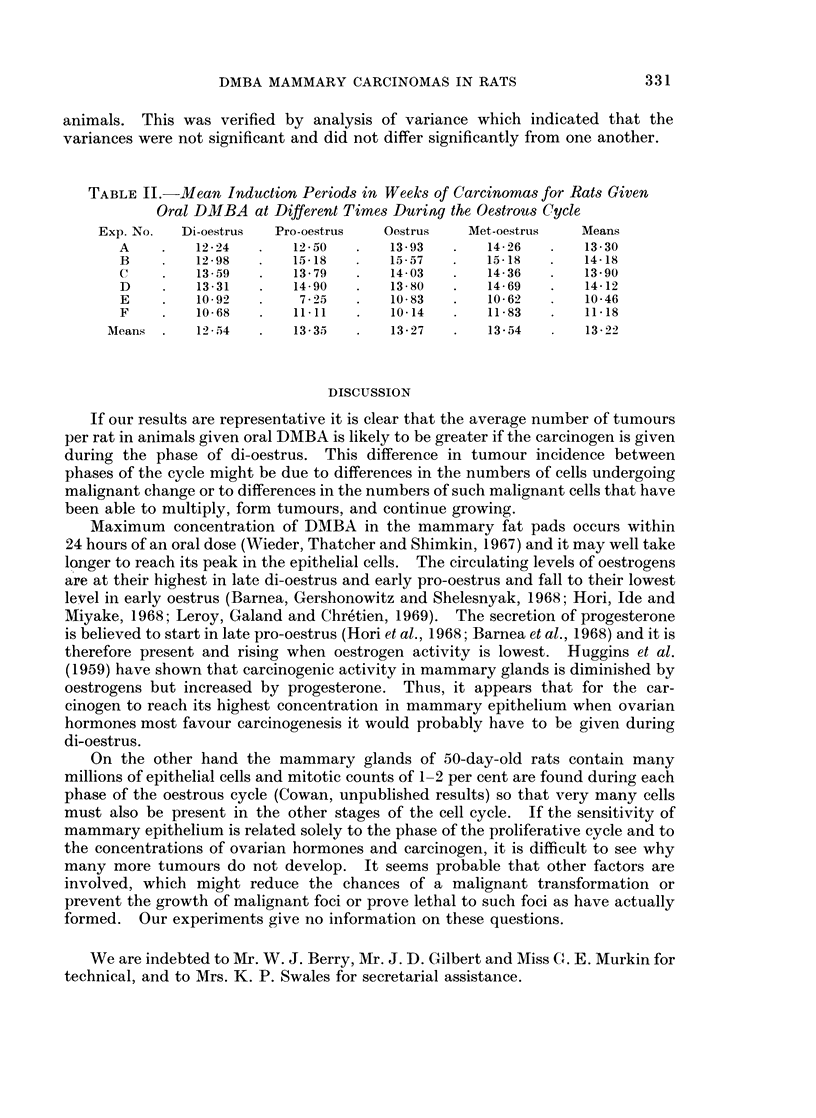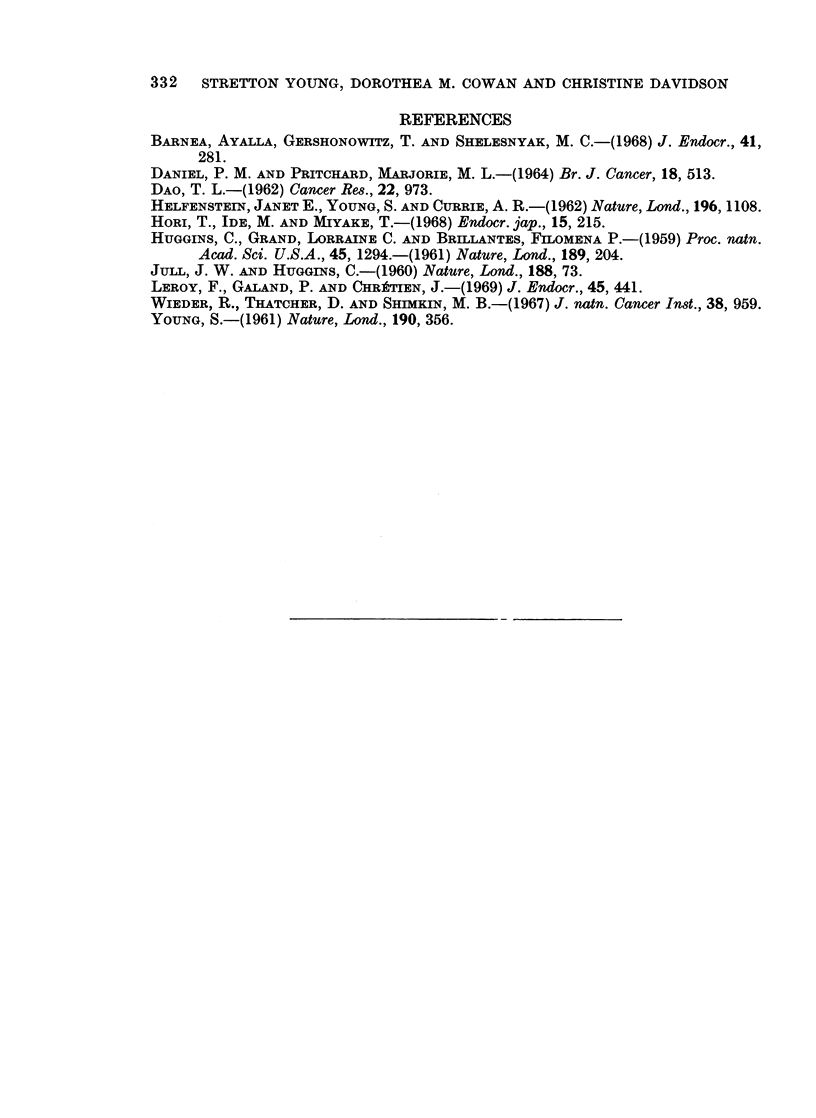# The Production of Mammary Carcinomas in Rats by 9,10-Dimethyl-1,2-Benzanthracene and its Relationship to the Oestrous Cycle

**DOI:** 10.1038/bjc.1970.38

**Published:** 1970-06

**Authors:** Stretton Young, Dorothea M. Cowan, Christine Davidson

## Abstract

Sprague-Dawley rats aged 50 days were given single oral doses of the carcinogen, DMBA.

Rats receiving the carcinogen at the same stage of the oestrous cycle were grouped together and mammary tumour production was compared between these groups.

When the carcinogen was given during di-oestrus the mean number of tumours per animal was significantly greater than when it was given at other stages in the oestrous cycle. There was considerable variation in total tumour yield from one batch of animals to another.


### THE PRODUCTION OF MAMMARY CARCINOMAS IN RATS BY 9,10-DIMETHYL-1,2-BENZANTHRACENE AND ITS RELATIONSHIP TO THE OESTROUS CYCLE

STRETTON YOUNG, DOROTHEA M. COWAN AND CHRISTINE DAVIDSON

*From the Department of Pathology, Imperial Cancer Research Fund, Lincoln's Inn Fields, London W.C.2*

Received for publication January 21, 1970

**SUMMARY.—Sprague-Dawley rats aged 50 days were given single oral doses of the carcinogen, DMBA.**

**Rats receiving the carcinogen at the same stage of the oestrous cycle were grouped together and mammary tumour production was compared between these groups.**

**When the carcinogen was given during di-oestrus the mean number of tumours per animal was significantly greater than when it was given at other stages in the oestrous cycle. There was considerable variation in total tumour yield from one batch of animals to another.**

IN any batch of rats given a single oral dose of 9,10-dimethyl-1,2-benzanthracene (DMBA) for the production of mammary carcinomas some rats will develop several tumours, others only a few and some may develop no tumours at all.

The yield of tumours from such rats is greatly reduced by castration but this can be reversed by replacement therapy using ovarian grafts (Dao, 1962). In certain doses oestradiol given to intact rats depresses, and progesterone enhances, mammary tumour production (Huggins, Grand and Brillantes, 1959, 1961). Tumour production is diminished in hypophysectomized animals but this effect also can be partly reversed by replacement of appropriate hormones (Young, 1961). Tumour production is reduced in rats made hypothyroid surgically (Jull and Huggins, 1960) or chemically (Helfenstein, Young and Currie, 1962) or made hyperthyroid by relatively large doses of l-thyroxine (Jull and Huggins, 1960).

There is thus ample evidence that hormones which affect mammary epithelium, plays a large part in determining the susceptibility of rats to orally administered carcinogens. It seemed possible therefore that variations in response might be related to cyclical variations in circulating oestrogens and hence to the phase of the oestrous cycle during which the carcinogen was given.

Experiments were set up to investigate this question and this paper records our results.

### MATERIALS AND METHODS

The rats were virgin female Sprague-Dawley. They were Caesarean derived, free from most normal pathogens (SPF) and random bred in the laboratories of the Imperial Cancer Research Fund. They were obtained at about 28 days of age, were housed 5 to a cage and were kept in an ambient temperature of about 70° F. (21° C.) with the lighting controlled to give equal periods of light and darkness.

A pelleted diet (formula GR3E, manufactured by Messrs. Dicksons of Ware) and water were both allowed *ad libitum.*

From about the 43rd day of age vaginal smears were taken at the same time on each day for 5 days per week and for 2 consecutive weeks. These were stained with Pasini's stain and were used to determine the stage of the oestrous cycle on the 50th day of age when each rat was given by stomach tube 2 ml. of corn oil containing 30 mg. of DMBA. Four weeks later weekly examinations for the presence of tumours were started. When the tumours had grown to about 1 cm. diameter they were removed under anaesthetic and slices were fixed in Bouin's fluid. They were embedded in paraffin wax and histological sections were cut and stained with haematoxylin and eosin. All tumours were examined histologically by one of us (S.Y.). Six experiments were set up in this way with a total of 296 rats. The experiments were assessed 20 weeks after the carcinogen was given when all surviving animals were killed.

### RESULTS

Two hundred and sixty-one rats survived to the end of the experiments and 199 of them developed mammary tumours. Out of the 987 tumours produced 965 had the histology of adenocarcinomas and 22 were fibroadenomas.

The experiments were intended to examine the frequency with which mammary adenocarcinomas developed under certain circumstances. Towards the end of the experiments very few new carcinomas were being detected, whereas fibroadenomas were found with increasing frequency. It is known (Daniel and Pritchard, 1964) that the induction time of fibroadenomas is much longer than that of carcinomas. Since the peak of fibroadenoma production was not reached these tumours have not been included in our tables.

Details of our results are given in Table I which shows:

(a) The number of animals at risk for each experiment and for each stage of the cycle.

(b) The numbers of rats developing carcinomas for each experiment and for each stage of the cycle.

(c) The numbers of carcinomas produced by rats at risk for each experiment and for each stage of the cycle.

The percentage of rats which developed mammary adenocarcinomas was comparatively uniform throughout the various stages of the oestrous cycle ($P$ is not significant), on the other hand the total number of carcinomas observed was higher than the expected value in the di-oestrous group and this difference was statistically significant, $P < 0 \cdot 001$. Similarly a comparison of the carcinomas produced by different experimental batches of animals showed considerable departure from the expected yield and these differences also were highly significant ($P < 0 \cdot 001$).

Analysis of variance was carried out on (a) the proportion of rats developing carcinomas and (b) the mean numbers of tumours per rat at risk for all experiments and for all stages of the cycle. Variance was almost equally divided between stages of the cycle and batches of animals, each was significant, the former being slightly more so than the latter.

The mean induction periods of carcinomas induced in these experiments are presented in Table II. From inspection it did not appear that the induction period was affected by the stage of the cycle any more than by different batches of

TABLE I.—*Numbers of Rats Affected by Carcinomas (Ca.) and Total Numbers of Carcinomas Induced After Giving DMBA at Different Times During the Oestrous Cycle*

| Exp. no. | Di-oestrus | | | Pro-oestrus | | | Oestrus | | | Met-oestrus | | | Totals | | | % Rats with Ca. |
|---|---|---|---|---|---|---|---|---|---|---|---|---|---|---|---|---|
| | Rats at risk | Rats with Ca. | No. of Ca. | Rats at risk | Rats with Ca. | No. of Ca. | Rats at risk | Rats with Ca. | No. of Ca. | Rats at risk | Rats with Ca. | No. of Ca. | Rats at risk | Rats with Ca. | No. of Ca. | |
| A | 15 | 11 | 54 | 9 | 6 | 18 | 9 | 7 | 40 | 12 | 7 | 49 | 45 | 31 | 161 | 68·9 |
| B | 19 | 16 | 95 | 11 | 8 | 56 | 4 | 4 | 14 | 12 | 10 | 39 | 46 | 38 | 204 | 82·6 |
| C | 6 | 5 | 55 | 16 | 14 | 68 | 9 | 8 | 35 | 14 | 9 | 47 | 45 | 36 | 205 | 80·0 |
| D | 12 | 10 | 70 | 11 | 9 | 40 | 5 | 3 | 10 | 10 | 10 | 52 | 38 | 32 | 172 | 84·2 |
| E | 7 | 5 | 25 | 6 | 3 | 4 | 15 | 8 | 19 | 15 | 9 | 26 | 43 | 25 | 74 | 58·1 |
| F | 11 | 10 | 51 | 10 | 8 | 19 | 7 | 6 | 14 | 16 | 13 | 65 | 44 | 37 | 149 | 84·1 |
| Totals | 70 | 57 | 350 | 63 | 48 | 205 | 49 | 36 | 132 | 79 | 58 | 278 | 261 | 199 | 965 | |
| % Rats with Ca. | | 81·4 | | | 76·2 | | | 73·5 | | | 73·4 | | | | | 76·2 |

animals. This was verified by analysis of variance which indicated that the variances were not significant and did not differ significantly from one another.

TABLE II.—*Mean Induction Periods in Weeks of Carcinomas for Rats Given Oral DMBA at Different Times During the Oestrous Cycle*

| Exp. No. | Di-oestrus | Pro-oestrus | Oestrus | Met-oestrus | Means |
|---|---|---|---|---|---|
| A | 12·24 | 12·50 | 13·93 | 14·26 | 13·30 |
| B | 12·98 | 15·18 | 15·57 | 15·18 | 14·18 |
| C | 13·59 | 13·79 | 14·03 | 14·36 | 13·90 |
| D | 13·31 | 14·90 | 13·80 | 14·69 | 14·12 |
| E | 10·92 | 7·25 | 10·83 | 10·62 | 10·46 |
| F | 10·68 | 11·11 | 10·14 | 11·83 | 11·18 |
| Means | 12·54 | 13·35 | 13·27 | 13·54 | 13·22 |

### DISCUSSION

If our results are representative it is clear that the average number of tumours per rat in animals given oral DMBA is likely to be greater if the carcinogen is given during the phase of di-oestrus. This difference in tumour incidence between phases of the cycle might be due to differences in the numbers of cells undergoing malignant change or to differences in the numbers of such malignant cells that have been able to multiply, form tumours, and continue growing.

Maximum concentration of DMBA in the mammary fat pads occurs within 24 hours of an oral dose (Wieder, Thatcher and Shimkin, 1967) and it may well take longer to reach its peak in the epithelial cells. The circulating levels of oestrogens are at their highest in late di-oestrus and early pro-oestrus and fall to their lowest level in early oestrus (Barnea, Gershonowitz and Shelesnyak, 1968; Hori, Ide and Miyake, 1968; Leroy, Galand and Chrétien, 1969). The secretion of progesterone is believed to start in late pro-oestrus (Hori *et al.*, 1968; Barnea *et al.*, 1968) and it is therefore present and rising when oestrogen activity is lowest. Huggins *et al.* (1959) have shown that carcinogenic activity in mammary glands is diminished by oestrogens but increased by progesterone. Thus, it appears that for the carcinogen to reach its highest concentration in mammary epithelium when ovarian hormones most favour carcinogenesis it would probably have to be given during di-oestrus.

On the other hand the mammary glands of 50-day-old rats contain many millions of epithelial cells and mitotic counts of 1–2 per cent are found during each phase of the oestrous cycle (Cowan, unpublished results) so that very many cells must also be present in the other stages of the cell cycle. If the sensitivity of mammary epithelium is related solely to the phase of the proliferative cycle and to the concentrations of ovarian hormones and carcinogen, it is difficult to see why many more tumours do not develop. It seems probable that other factors are involved, which might reduce the chances of a malignant transformation or prevent the growth of malignant foci or prove lethal to such foci as have actually formed. Our experiments give no information on these questions.

We are indebted to Mr. W. J. Berry, Mr. J. D. Gilbert and Miss G. E. Murkin for technical, and to Mrs. K. P. Swales for secretarial assistance.

### REFERENCES

BARNEA, AYALLA, GERSHONOWITZ, T. AND SHELESNYAK, M. C.—(1968) *J. Endocr.*, **41**, 281.

DANIEL, P. M. AND PRITCHARD, MARJORIE, M. L.—(1964) *Br. J. Cancer*, **18**, 513.

DAO, T. L.—(1962) *Cancer Res.*, **22**, 973.

HELFENSTEIN, JANET E., YOUNG, S. AND CURRIE, A. R.—(1962) *Nature, Lond.*, **196**, 1108.

HORI, T., IDE, M. AND MIYAKE, T.—(1968) *Endocr. jap.*, **15**, 215.

HUGGINS, C., GRAND, LORRAINE C. AND BRILLANTES, FILOMENA P.—(1959) *Proc. natn. Acad. Sci. U.S.A.*, **45**, 1294.—(1961) *Nature, Lond.*, **189**, 204.

JULL, J. W. AND HUGGINS, C.—(1960) *Nature, Lond.*, **188**, 73.

LEROY, F., GALAND, P. AND CHRÉTIEN, J.—(1969) *J. Endocr.*, **45**, 441.

WIEDER, R., THATCHER, D. AND SHIMKIN, M. B.—(1967) *J. natn. Cancer Inst.*, **38**, 959.

YOUNG, S.—(1961) *Nature, Lond.*, **190**, 356.